# Soluble Klotho, a biomarker and therapeutic strategy to reduce bronchopulmonary dysplasia and pulmonary hypertension in preterm infants

**DOI:** 10.1038/s41598-020-69296-1

**Published:** 2020-07-23

**Authors:** Sunil Batlahally, Andrew Franklin, Andreas Damianos, Jian Huang, Pingping Chen, Mayank Sharma, Joanne Duara, Divya Keerthy, Ronald Zambrano, Lina A. Shehadeh, Eliana C. Martinez, Marissa J. DeFreitas, Shathiyah Kulandavelu, Carolyn L. Abitbol, Michael Freundlich, Rosemeire M. Kanashiro-Takeuchi, Augusto Schmidt, Merline Benny, Shu Wu, Karen K. Mestan, Karen C. Young

**Affiliations:** 1 Department of Pediatrics, University of Miami Miller School of Medicine, Miami, FL USA; 2 Batchelor Children’s Research Institute, University of Miami Miller School of Medicine, 1580 NW 10th Avenue, RM-345, Miami, FL USA; 3 The Interdisciplinary Stem Cell Institute, University of Miami Miller School of Medicine, Miami, FL USA; 4 Department of Molecular and Cellular Pharmacology, University of Miami Miller School of Medicine, Miami, FL USA; 5 Ann & Robert H. Lurie Children’s Hospital of Chicago, Northwestern University Feinberg School of Medicine, Chicago, IL USA

**Keywords:** Paediatric research, Preclinical research

## Abstract

Preterm infants with bronchopulmonary dysplasia (BPD) and pulmonary hypertension (PH) have accelerated lung aging and poor long-term outcomes. Klotho is an antiaging protein that modulates oxidative stress, angiogenesis and fibrosis. Here we test the hypothesis that decreased cord Klotho levels in preterm infants predict increased BPD–PH risk and early Klotho supplementation prevents BPD-like phenotype and PH in rodents exposed to neonatal hyperoxia. In experiment 1, Klotho levels were measured in cord blood of preterm infants who were enrolled in a longitudinal cohort study. In experiment 2, using an experimental BPD–PH model, rat pups exposed to room air or hyperoxia (85% O_2_) were randomly assigned to receive every other day injections of recombinant Klotho or placebo. The effect of Klotho on lung structure, PH and cardiac function was assessed. As compared to controls, preterm infants with BPD or BPD–PH had decreased cord Klotho levels. Early Klotho supplementation in neonatal hyperoxia-exposed rodents preserved lung alveolar and vascular structure, attenuated PH, reduced pulmonary vascular remodeling and improved cardiac function. Together, these findings have important implications as they suggest that perinatal Klotho deficiency contributes to BPD–PH risk and strategies that preserve Klotho levels, may improve long-term cardiopulmonary outcomes in preterm infants.

## Introduction

Bronchopulmonary dysplasia (BPD) is a multi-factorial disease which affects premature infants^1^. It is characterized by alterations in lung development, vascular remodeling and lung dysfunction that range the spectrum of mild to severe and even fatal disease. Preterm infants with BPD often grow poorly^2^ and in the most severe cases develop pulmonary hypertension (PH)^3^, and myocardial dysfunction^4,5^. According to data from the National Institute of Child Health and Human Development, during the years 2003 to 2007, the incidence of BPD in infants less than 29 weeks gestational age was 42%^6^ with an increasing trend over the past 10 years^7^. Not only does BPD increase infant mortality but survivors have cardiopulmonary dysfunction extending long into adulthood^8^.

Various prenatal and postnatal factors contribute to the development of BPD and its sequelae. Infants with placental vascular malperfusion are at an increased risk for BPD and PH^9^ while hyperoxia^10^, infection^11^ and poor nutrition^12^ are among the many postnatal insults which increase lung oxidative stress and create a pro-inflammatory milieu. This suboptimal intrauterine and neonatal environment, during a pivotal period of organogenesis, alters key developmental signaling pathways, disrupts intercellular communication, induces cellular apoptosis and promotes early lung aging.

Klotho is an anti-aging gene first identified by Kuro-o et al. in 1997^13^. Homozygous Klotho deficient mice have premature aging, spontaneous fibrosis, pulmonary emphysema and vascular dysfunction, while mice that over-express Klotho have extended life span and relative protection from cardiopulmonary injury^13^. Klotho is primarily expressed in the kidneys and choroid plexus^13^, but it is also present in the placenta^14^, lungs, parathyroid glands and heart^13^. Klotho exists in two main forms: full-length transmembrane Klotho and soluble Klotho, the latter being formed by proteolytic cleavage of membrane Klotho.

The role of soluble Klotho in disease and repair is not fully understood. Emerging evidence suggest that soluble Klotho exhibits humoral activity as Klotho deficient mice have functional deficits in organs which do not express Klotho^15^. Other reports demonstrate that soluble Klotho may act as an on demand co-receptor for fibroblast growth factor 23 (FGF-23)^16^ or it may act independently of FGF23 and interact with monosialogangliosides in lipid rafts of the plasma membrane, altering their lipid organization and modulating biological pathways that regulate oxidative stress^17,18^, apoptosis^19^, stem cell renewal^20^, fibrogenesis^21^ and angiogenesis^22^. Soluble Klotho reduces oxidative stress by promoting forkhead transcription factor activation and nuclear translocation, leading to upregulation of manganese superoxide dismutase (MnSoD)^23^. It also suppresses fibrosis by binding to type II transforming growth factor-beta 1 (TGF-β) receptor, preventing TGF-β binding and inhibiting SMAD 2/3 signaling^21^.

Although multiple age-related morbidities exhibit reduced Klotho levels^24,25^, little is known about the role of Klotho in neonatal diseases. Intra-amniotic infection and smoking is associated with decreased maternal plasma Klotho concentrations^26^ and pregnancies complicated by pre-eclampsia and small for gestational age infants exhibit lower soluble Klotho levels^27,28^. Interestingly, while the latter perinatal insults contribute to BPD and poor long-term outcomes, to the best of our knowledge, there are no published data evaluating the relationship between cord Klotho levels and BPD or PH. Moreover, whether early administration of Klotho can reduce BPD, PH and long-term myocardial dysfunction has not been previously explored.

In the present study, we tested the hypothesis that decreased cord Klotho levels in preterm infants is associated with increased BPD–PH risk and that early Klotho supplementation decreases oxidative stress, suppresses fibrosis, improves lung vascular development and prevents BPD-like phenotype and PH in rodents exposed to neonatal hyperoxia. We demonstrate in a cohort of prospectively followed preterm infants that decreased cord blood Klotho concentration is associated with BPD and BPD–PH. In addition, using an in vivo preclinical model of severe BPD–PH, we show that early administration of soluble Klotho improves lung vascular development, attenuates PH, and reduces myocardial dysfunction. In vitro, we demonstrate that Klotho treatment of hyperoxia-exposed human pulmonary artery endothelial cells (HPAECs) increases cell survival, proliferation and capillary-tube formation. Together, these findings have important implications as they suggest that perinatal Klotho deficiency may increase the susceptibility to BPD and PH in preterm infants. Moreover, we speculate that strategies which increase soluble Klotho levels, may improve cardiopulmonary outcomes in preterm neonates.

## Methods

### Human cord blood and placenta study

#### Study design and patient sample

We performed a nested case–control study of infants < 29 weeks GA from an ongoing prospective longitudinal cohort study at Prentice Women’s Hospital in Chicago, IL, USA from 2008-present. Eligible patients of the parent study are live births with 23–41 completed weeks of gestation with available cord blood. For this study, all infants less than 29 weeks gestation from 2017–2018 with available cord blood were included. Comprehensive maternal and infant data were collected. Infants were classified as BPD using the NIH consensus definitions. BPD–PH was defined using echocardiography parameters at 36 weeks corrected gestational age as previously published in this cohort^9^. This study was approved by the Institutional Review Board of Northwestern University and all methods were carried out in accordance with approved guidelines. Informed consent was obtained from all mothers prior to participation.

#### Cord blood Klotho measurement

Venous cord blood was collected at birth by trained delivery staff. Samples were centrifuged for 10 min at − 4 °C at 3,000 rpm. Plasma was removed by pipetting and aliquots were archived at − 80 °C. Samples were retrieved from the Prentice Birth Cohort Repository and thawed on ice. Only samples without hemolysis were included. Soluble Klotho levels were measured via enzyme-linked immunoassay (Immuno-Biological Laboratories, MN) using the protocol written by the manufacturer using 1:4 dilutions in duplicate.

### Experimental BPD–PH model

#### Animals

Pregnant Sprague Dawley rats were purchased from Charles River Laboratories (Wilmington, MA). Rats were treated according to National Institute of Health guidelines for the use and care of laboratory animals following approval of the study protocol by the University of Miami Animal Care and Use Committee.

#### Experimental BPD and PH protocol

Sprague Dawley pups assigned to normoxia (21% O_2_) or hyperoxia (85% O_2_), were randomly assigned to receive intraperitoneal (IP) injections of recombinant Klotho 30 mcg/kg ( Cat# 1819-KL; R&D Systems; Minneapolis, MN), or phosphate buffered saline as placebo (PL) every other day from postnatal day 1 to 21. This dose was based on pilot studies in our laboratory. Oxygen exposure was achieved in a Plexiglas chamber by a flow-through system and the oxygen level inside the chamber was monitored daily with a Maxtec Oxygen Analyzer (Model OM-25; Maxtec, Salt Lake City, Utah). Dams were rotated every 48 h between hyperoxia and normoxia chambers to prevent oxygen-induced damage to their lungs. Litter size was adjusted to 10–12 pups to control for the effect of litter size on nutrition and growth. The animals were recovered in normoxia for three additional weeks prior to sacrifice. Transthoracic echocardiography, lung morphometric and molecular studies were performed at postnatal day 42.

#### Lung morphometric analysis

Lung alveolarization was evaluated as previously described^29–31^. Briefly, a 23-gauge silastic catheter was introduced through the right ventricular wall and advanced into the pulmonary artery. The catheter was connected to a reservoir containing phosphate buffered saline (PBS). This solution was delivered at an air driven pressure of 40 cmH_2_O for 5 min and then the left atrium was punctured after distension. After completion of the vascular perfusion, a vascular fixative containing 4% paraformaldehyde was delivered at the same pressure and duration. The airways were perfused through the trachea at a transpulmonary pressure of 15 cmH_2_O for 5 min with 4% paraformaldehyde. The lungs were fixed overnight in 4% paraformaldehyde and then transferred to 70% ethanol and then subsequently embedded in paraffin. Serial Sects. (5 μm) thick were taken from the upper and lower lobes and stained with hematoxylin and eosin. Images from five randomly selected, non-overlapping parenchymal fields were acquired from lung sections of each animal using an Olympus Qcolor 3 color camera interfaced with a light microscope (Model Leica DMI 4000B) at 20X magnification. Alveolarization was determined by calculating mean linear intercept (MLI) and septal thickness. Mean linear intercept (MLI) was calculated by determining average distance between intersects of alveolar septal tissue and a superimposed counting grid^30^. Septal thickness was assessed on hematoxylin and eosin stained lung sections by averaging 100 measurements per 10 representative fields at 40X magnification.

#### Pulmonary vascular density

Lung vascular density was evaluated as previously described^29,30^. Briefly, sections were de-paraffinized, rehydrated, and stained with polyclonal rabbit anti-human Von Willebrand Factor (vWF, 1:50; Dako Corp, Carpintaria, CA). The number of vessels (20–50 μm diameter) per high power field (HPF) was quantified in five randomly selected, non-overlapping, parenchymal fields from lung sections of each animal.

#### Pulmonary vascular remodeling

Pulmonary vascular remodeling was evaluated as previously described^30^. Briefly, paraffin embedded sections were stained with polyclonal rabbit anti-human vWF and monoclonal mouse anti-α-smooth muscle actin (α-SMA: 1:500, Sigma-Aldrich; St. Louis, MO). Medial wall thickness (MWT) of partially and fully muscular arteries (20–50 μm) was determined by using the formula: 2(MT) × 100/ED, where MT is the distance between the internal and external boundaries of the α-SMA layer and ED is the external diameter. The percentage of peripheral pulmonary vessels (< 50 µm in diameter) stained with α-SMA > 50% of the circumference was determined from ten random images on each lung section and all analyses were performed by a blinded observer.

#### Transthoracic echocardiography

Rats underwent echocardiography using a Vevo2100 Imaging System (Fujifilm Visual Sonics, Inc. Ontario, Canada). Echocardiography was performed under anesthesia via isoflurane inhalation (2% to 3%), controlled heart rates (≥ 400 bpm), and core body temperatures (37 ± 1 ℃). Left ventricular (LV) ejection fraction was assessed using M-mode and tricuspid annular plane systolic excursion (TAPSE) was measured using a modified 4-chamber view. Pulmonary artery Doppler was used to assess pulmonary artery acceleration time (PAAT) and pulmonary artery ejection time (PET). The average of 3–5 representative cardiac cycles was used to calculate values, using Vevo LAB Software (version 3.2.0).

#### Nitrotyrosine immunostaining

Paraffin embedded sections of lungs and right ventricles were de-paraffinized, rehydrated and stained with mouse monoclonal antibody to 3-Nitrotyrosine (1:1,000, Cat# ab125106, Abcam, Cambridge, MA). 3-Nitrotyrosine expression (brown) was qualitatively determined by examining slides under a light microscope (Model Leica DMI 4000B) at 40X magnification.

#### Masson’s trichrome staining

Paraffin embedded sections of lungs and right ventricles were de-paraffinized, rehydrated and stained with Masson’s trichrome as per manufacturer’s instructions (Cat # HT15-1KT, Sigma-Aldrich). Fibrosis was qualitatively determined by examining slides under a light microscope (Model Leica DMI 4000B) at 40X magnification.

#### Western blot analysis

The protein expression of α-Klotho, MnSOD, TGF-β, and CC3 were determined by Western blot analysis as previously described^29^. The polyclonal antibodies for α-Klotho (Cat# 154163; 1:1,000), MnSOD (Cat# 13533; 1:2,500) and TGF-β (Cat# 179695; 1:500) were obtained from Abcam (Cambridge, MA). CC3 polyclonal antibody (Cat # 9664; 1:500) was obtained from Cell Signaling Technology (Beverly, MA). Total protein was extracted from frozen lung, RV and LV tissues with a RIPA buffer according to the manufacturer’s protocol (Santa Cruz, Dallas, TX). Protein concentration was measured by BCA protein assay using a commercial kit from Pierce Biotechnology Inc. (Rockford, IL). Lung, RV and LV lysate (50 μg/sample) were fractionated by SDS-PAGE on 4–20% Mini-Protean Tris–Glycine Extended Precast Protein Gel (Bio-Rad, Hercules, CA) and transferred to nitrocellulose membranes (Amersham, Piscataway, NJ). Immunodetection was performed by incubating the membranes with the primary antibodies diluted in blocking buffer overnight at 4ºC and then for 1 h at room temperature with horseradish peroxidase-conjugated secondary antibodies. Antibody bound protein was detected using enhanced chemiluminescence methodology (Amersham). Band intensity was quantified with Quantity One Software (Bio-Rad, Hercules, CA), with β-Actin acting as the normalization protein (1:10,000; Sigma-Aldrich, St. Louis, MO) for lung samples and GAPDH as the normalization protein for heart samples (1:10,000; Santa Cruz Biotechnology, Santa Cruz, CA).

#### Real time RT-PCR

Lung Klotho gene expression was determined by real time RT-PCR. RNA from lung tissue was extracted (miRNeasy Mini Kit; Cat# 217004; Qiagen, Inc. Valencia, CA) and reverse transcribed (Superscript VIVILO Master Mix; Cat# 11766050, ThermoFisher, Cambridge, MA). Real time RT-PCR using TaqMan Fast Advanced Master Mix (Cat# 4444554, Applied Biosystems, Foster City, CA) was performed on an ABI Fast 7500 system (Applied Biosystems). Primers for Klotho (Cat# Rn0058012) and GAPDH (Cat# Rn99999916) were pre-developed by Applied Biosystems. The relative mRNA expression of Klotho was normalized to GAPDH expression.

#### ELISA

Rat Klotho serum concentration was determined by ELISA kit as per manufacturer instructions (LifeSpan Biosciences, Seattle, WA).

### HPAECs Culture

HPAECs (Cat # ATCCPCS-100–022, ATCC, Manassas, VA) were cultured to passage 3 to 6, plated in 100 mm dishes and serum starved for 48 h. Serum-starved HPAECs were treated with varying doses of recombinant human Klotho (Cat # 5334-KL-025; R&D Systems; 0.01–0.5 µg/ml) and cultured in normoxic (21% O_2_, 5% CO_2_) or hyperoxic (95% O_2_, 5% CO_2_) conditions for 24 h.

#### Matrigel assay

The effect of Klotho on capillary tube formation was determined by matrigel assay as previously described^32^. Capillary tube formation was assessed on growth factor reduced matrigel-coated wells (BD Biosciences, San Diego, CA). Bright field images were collected at 12 h. All experiments were done in triplicate and tube formation was assessed using Image J Angiogenesis Analyzer.

#### Cell viability

The effect of Klotho on HPAECs viability was determined by MTT assay (Cat # M5655, Sigma-Aldrich, Saint Louis, MO). HPAECs (1 × 10^4^ cells/well) cultured overnight in 96-well plates and treated with Klotho (0.01–0.5 µg/ml) were exposed to normoxia (21% O_2_, 5% CO_2_) or hyperoxia (95% O_2_, 5% CO_2_) for 24 h. Twenty microliters of MTT labeling solution was added 4 h before the end of the incubation. The plate was then incubated at 37 ºC for 5 min following which it was transferred to a plate reader (SpectraMax Plus 384 Microplate Reader; San Jose, CA) and absorbance was measured at a wavelength of 550 nm. The experiment was performed in triplicate.

#### TUNEL assay

Terminal deoxynucleotidyl transferase-mediated dUTP nick-end labeling (TUNEL) staining of HPAECs was performed using a TUNEL kit (Cat# C10617, Thermofisher) as per manufacturer instructions. Five randomly selected fields were photographed, and the number of apoptotic nuclei as well as the total number of nuclei was counted per HPF. The apoptotic index was obtained by means of the formula: number of apoptotic cells per field / total number of cells per field as previously described^31^.

### Statistical analysis

Human clinical data were analyzed using Kruskal–Wallis non-parametric testing when comparing median biomarker concentrations as distribution of Klotho was not normal by Shapiro–Wilk testing. Cord blood Klotho values are displayed as median [interquartile range]. Preclinical results reported as mean ± standard error of mean (SEM), were analyzed by two-way ANOVA with post-hoc Holm-Sidak test using Sigma Stat software. *P* values less than 0.05 were considered statistically significant.

## Results

### Human cord blood study

#### Clinical characteristics of the sample

The clinical data of 40 infants including (11 with BPD, 14 with BPD–PH, and 15 without either BPD or BPD–PH) are summarized in Table 1. Infants who developed BPD were significantly lower in gestational age at birth. Those who did not develop BPD or BPD–PH had significantly higher birth weight, although there was no difference in birth weight for gestational age. There were no differences between the groups in sex or maternal characteristics including maternal age, race, preterm labor, preeclampsia, mode of delivery, or rates of chorioamnionitis. Infants who developed BPD and BPD–PH had significantly lower 1 and 5 min Apgar scores.

**Table 1:** Demographics and clinical characteristics of the patient sample according to BPD and PH status.

	Control (n = 15)	BPD (n = 11)	BPD–PH (n = 14)	*P* value
**Infant characteristics**				
Gestational age (weeks)	27.5 ± 1	26.1 ± 1.5	27 ± 1	0.02
Birth weight (grams)	1,081 ± 197	940 ± 127	901 ± 173	0.02
Birth weight percentile	67 ± 24	73 ± 23	55 ± 29	NS
Male sex (%)	6 (40)	8 (73)	4 (29)	NS
**Maternal characteristics**				
Maternal age (years)	28.2 ± 7.2	31.1 ± 5.9	32.1 ± 6.2	NS
**Maternal race**				
Black	5 (33)	0	2 (14)	NS
White	5 (33)	6 (55)	9 (64)	
Asian	0	0	2 (14)	
Hispanic	4 (27)	5 (45)	1 (7)	
Other/unknown	1 (6)	0	0	
Preterm labor (%)	10 (67)	10 (91)	11 (79)	NS
Premature rupture of membranes (%)	6 (40)	5 (45)	4 (29)	NS
Prolonged rupture of membranes (%)	5 (33)	4 (36)	4 (29)	NS
Cesarean section (%)	8 (53)	9 (82)	12 (86)	NS
Antenatal steroids (%)	14 (93)	11 (100)	13 (93)	NS
Preeclampsia (%)	3 (20)	1 (9)	3 (21)	NS
Chrorioamnionitis (%)	3 (20)	1 (9)	1 (7)	NS
Non-reassuring fetal heart tones (%)	2 (13)	1 (9)	1 (7)	NS
Apgar 1 min (mean)	6 + 1.9	3.6 + 2.2	3.9 + 2.5	0.02
Apgar 5 min (mean)	7.9 ± 0.7	6.4 ± 1.4	5.7 ± 2.6	0.005

#### Decreased cord blood Klotho concentration in preterm infants is associated with BPD and BPD–PH

**Figure 1:**
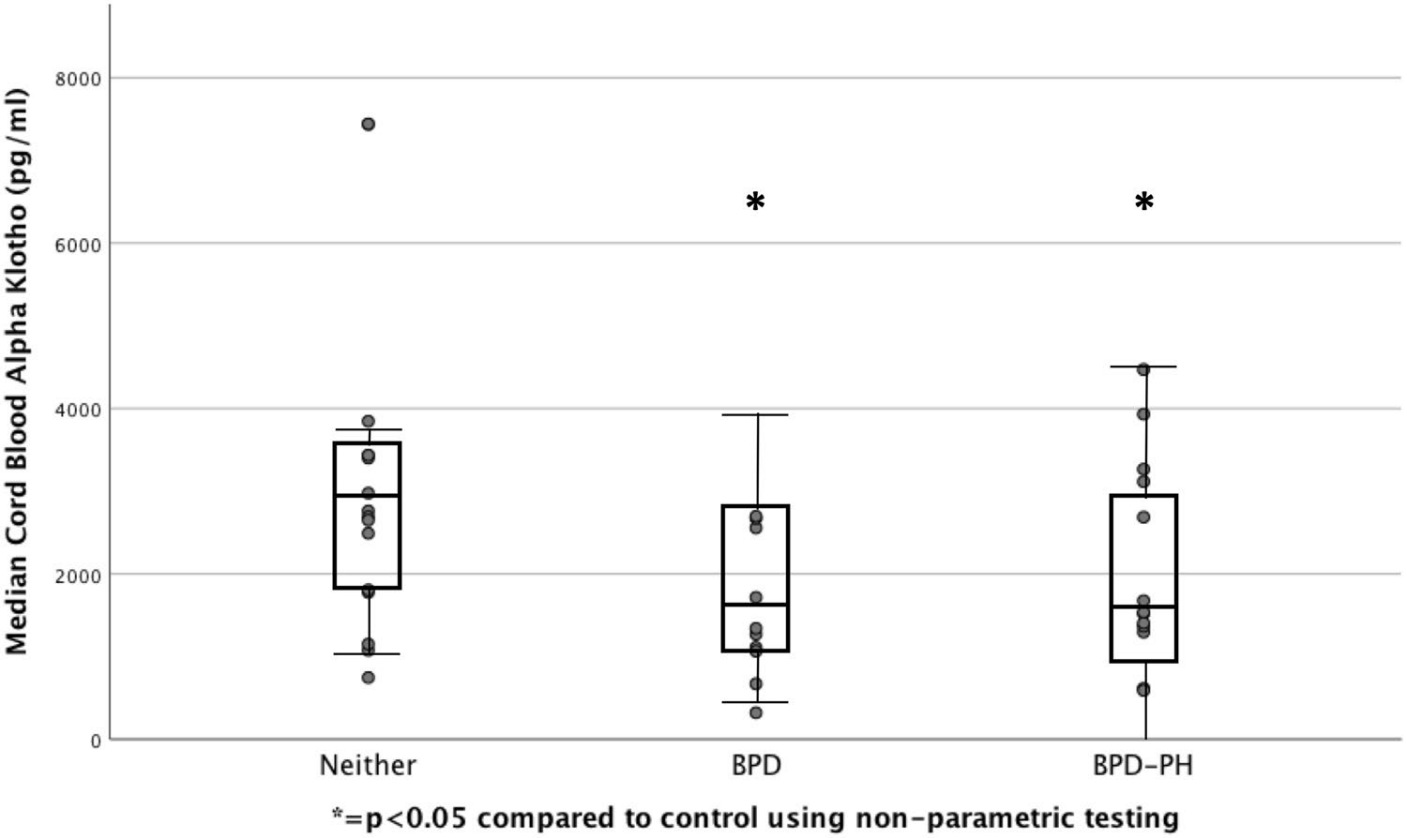
Cord Klotho levels in preterm infants. Significantly reduced cord blood Klotho concentration in preterm infants with BPD or BPD–PH as compared to control. *P < 0.05 compared to control using non-parametric testing.

Klotho concentration was decreased in cord blood of preterm infants who developed BPD and BPD–PH compared with controls (BPD = 1,306 pg/ml [967, 2585]; BPD–PH = 1529 pg/ml [1126, 3153]; and control = 2,726 pg/ml [1784, 3435], both *P* < 0.05 (Fig. 1).

### Experimental BPD–PH model

#### Neonatal hyperoxia decreases lung and circulating Klotho levels

**Figure 2:**
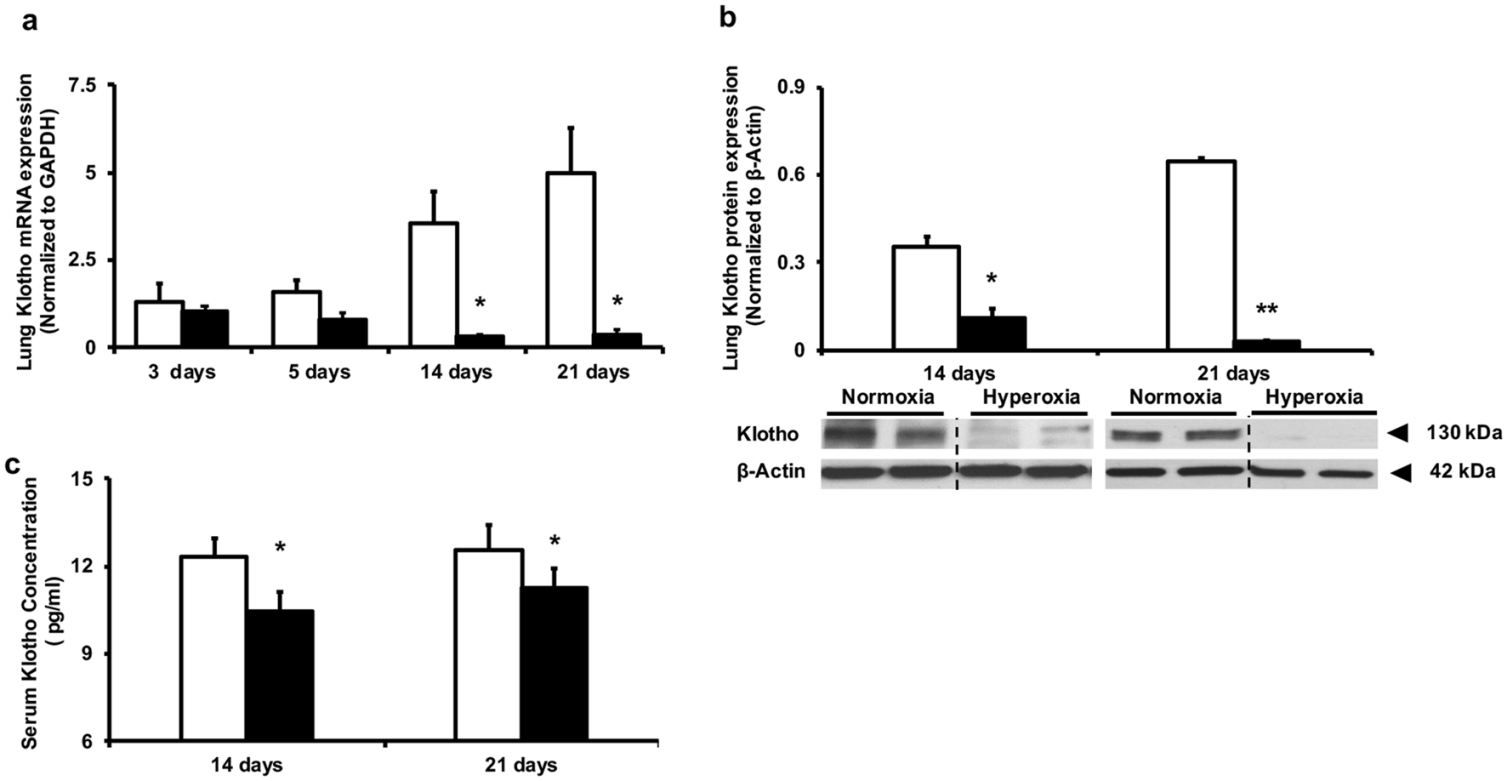
Neonatal hyperoxia exposure reduces lung and circulating Klotho levels. Decreased (**a**) lung Klotho mRNA and (**b**) protein expression in rats exposed to 14 and 21 days hyperoxia. Klotho gene and protein expression were normalized to GAPDH and β-Actin respectively. A representative Western blot is shown in the lower panel. (**c**) Reduced circulating Klotho levels in rodents exposed to 14 and 21 days hyperoxia. White bars indicate normoxia, and black bars indicate hyperoxia (data are mean ± SEM; **P < 0.001, *P < 0.05; normoxia vs hyperoxia; N = 4–5/group).

Given the multifactorial etiology of BPD and PH in preterm infants and the significant contribution of hyperoxia to the pathogenesis of these diseases, we evaluated the effect of neonatal hyperoxia on lung and circulating Klotho levels in our experimental BPD–PH model. Neonatal rat pups were exposed to room air or hyperoxia (85% O_2_) for 3, 5, 14 and 21 days and lung Klotho gene and protein expression along with circulating soluble Klotho levels were assessed. The mRNA Klotho gene expression in the lung tissue of neonatal rat pups exposed to hyperoxia for 3 days was comparable to that of the group exposed to normoxia (Fig. 2a). While Klotho levels trended to be lower in pups exposed to 5 days of hyperoxia, this was not statistically significant. Western blot analysis displayed similar band intensities of the Klotho protein levels in the lungs of animals following 3 or 5 days of either hyperoxia or normoxia (Fig. 2b). Of note, more prolonged exposure to 14 and 21 days of hyperoxia resulted in a significantly reduced lung Klotho gene and protein expression levels (Fig. 2a,b). Concurrent blood sampling at these two time points, days 14 and 21, revealed significantly lower serum levels of Klotho in the pups exposed to hyperoxia compared with Klotho levels in the normoxia group (Fig. 2c).

#### Early Klotho supplementation improves lung vascular development

**Figure 3:**
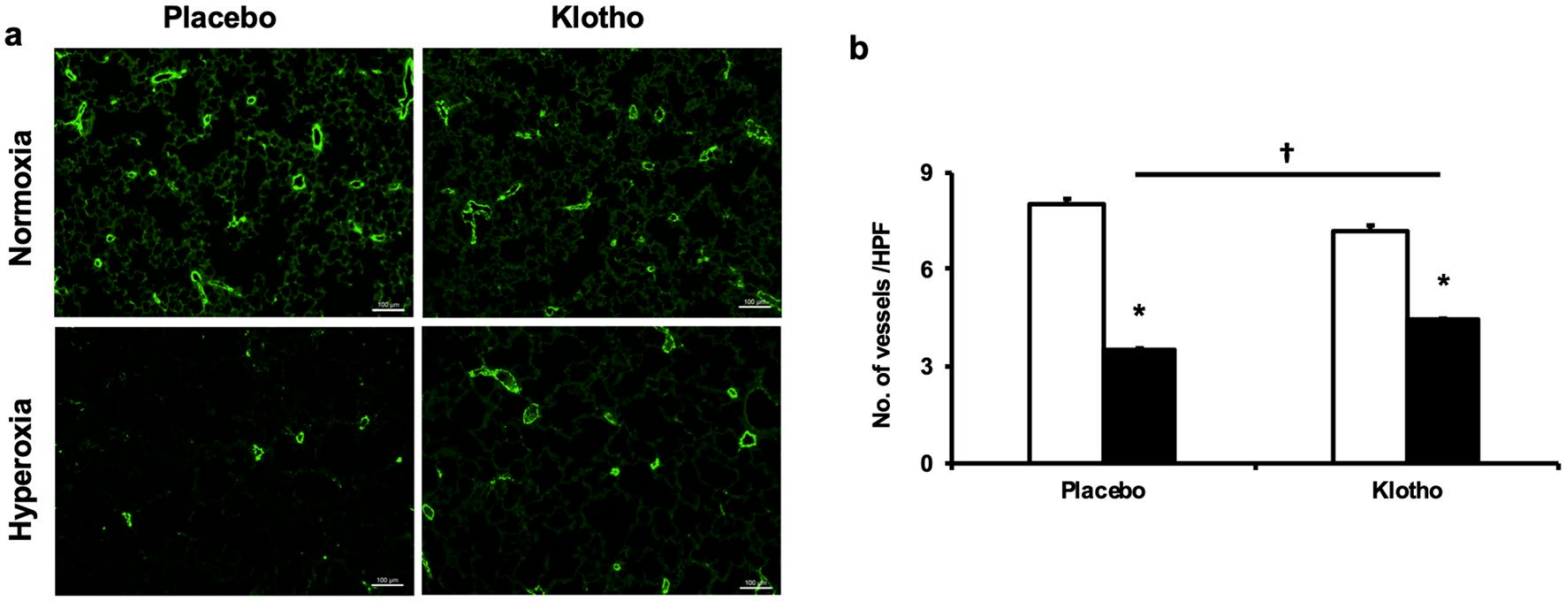
Klotho improves lung angiogenesis. (**a**) Lung sections stained with von Willebrand Factor (vWF) antibody showing improved lung vascular density in hyperoxia-exposed rats treated with Klotho. Original magnification × 20. Scale bars are 50 µm. (**b**) Histogram showing increased vascular density in Klotho treated hyperoxia-exposed rats (data are mean ± SEM; *P* < 0.05; * normoxia vs hyperoxia; † hyperoxia-PL vs hyperoxia–Klotho; N = 5–7/group). White bars indicate normoxia and black bars indicate hyperoxia.

Since disordered lung angiogenesis is a key component of BPD–PH, and soluble Klotho has vasculoprotective effects, we next evaluated whether early Klotho supplementation would preserve lung angiogenesis in our experimental BPD–PH model. Rat pups exposed to room air or hyperoxia (85% O_2_) from postnatal day (P) 1 to 21 were randomly assigned to receive every other day injections of recombinant Klotho or placebo (PL). The effect of Klotho on lung vascular development was assessed on P42. Lung sections were immunostained with vWF and the number of vessels (20–50 µm) per HPF quantified. PL-treated hyperoxia exposed rats had reduced lung vascular density, (8 ± 0.1 vs 3.5 ± 0.03 vessels/HPF; normoxia-PL vs hyperoxia-PL; *P* < 0.05; N = 4–5/group), but this significantly improved following early Klotho supplementation, (Fig. 3a,b).

#### Klotho treatment of hyperoxia-exposed human pulmonary artery endothelial cells (HPAECs)

**Figure 4:**
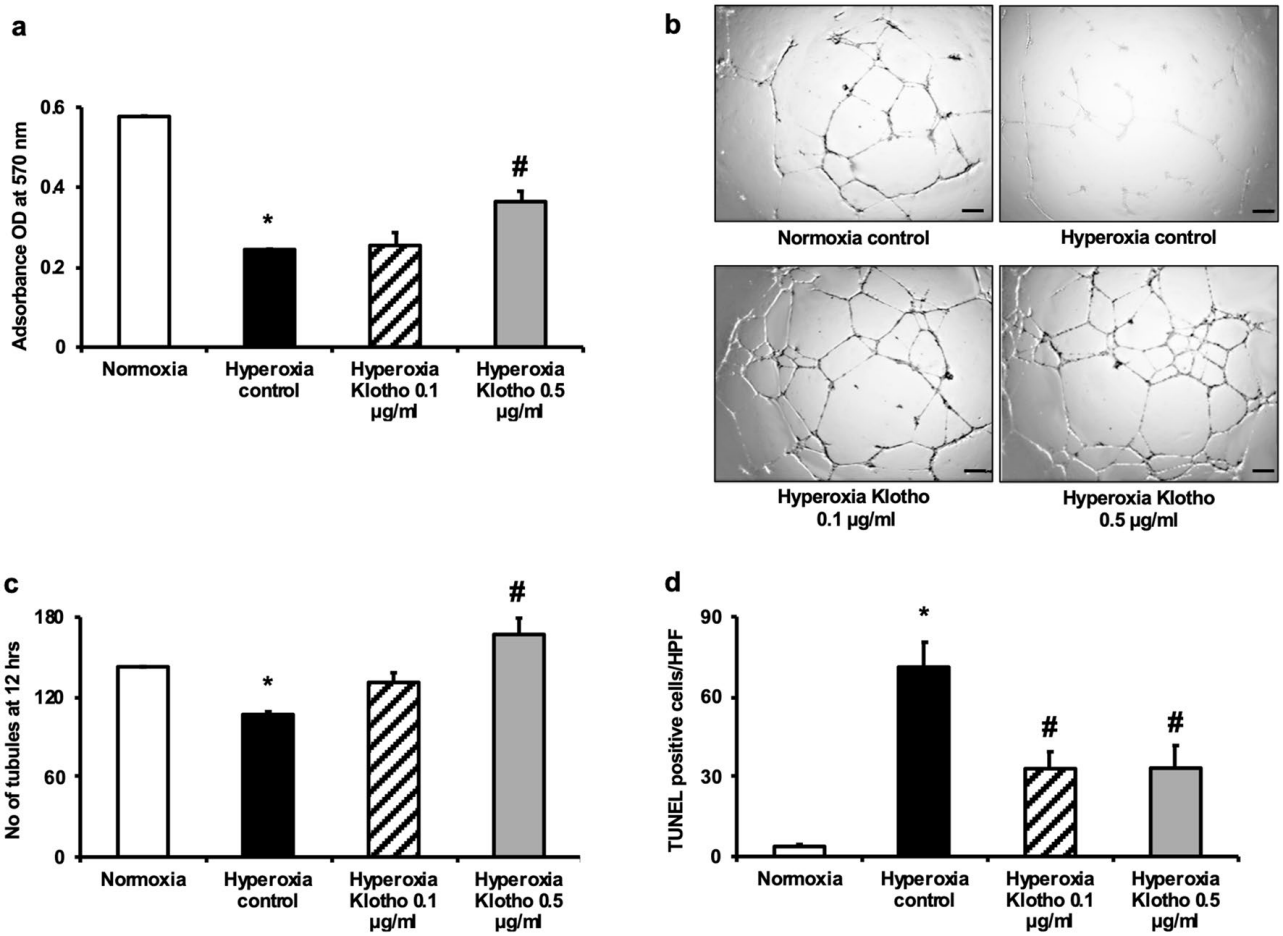
Klotho improves cell viability, capillary tube formation and survival in hyperoxia-exposed HPAECs. Treatment of hyperoxia-exposed HPAECs with recombinant Klotho increased (**a**) cell viability (**b**, **c**) capillary tube formation and (**d**) survival (data are mean ± SEM; *P* < 0.05; *normoxia vs hyperoxia-control; # hyperoxia-control vs hyperoxia-Klotho 0.1 or 0.5 ug/ml; all experiments were performed in triplicate). Original magnification × 2.5.

Next, in order to confirm that soluble Klotho acts directly on the pulmonary vasculature, we exposed HPAECs to hyperoxia and treated the cells with varying doses of recombinant Klotho. Whereas hyperoxia-exposed HPAECs had significantly decreased cell viability, capillary tube formation and survival (all *P* < 0.05 vs normoxia), treatment with Klotho increased HPAECs viability, number of capillary tube like structures and HPAECs survival (all *P* < 0.05 vs hyperoxia-control) (Fig. 4a–d).

#### Early Klotho supplementation attenuates PH and vascular remodeling

**Figure 5:**
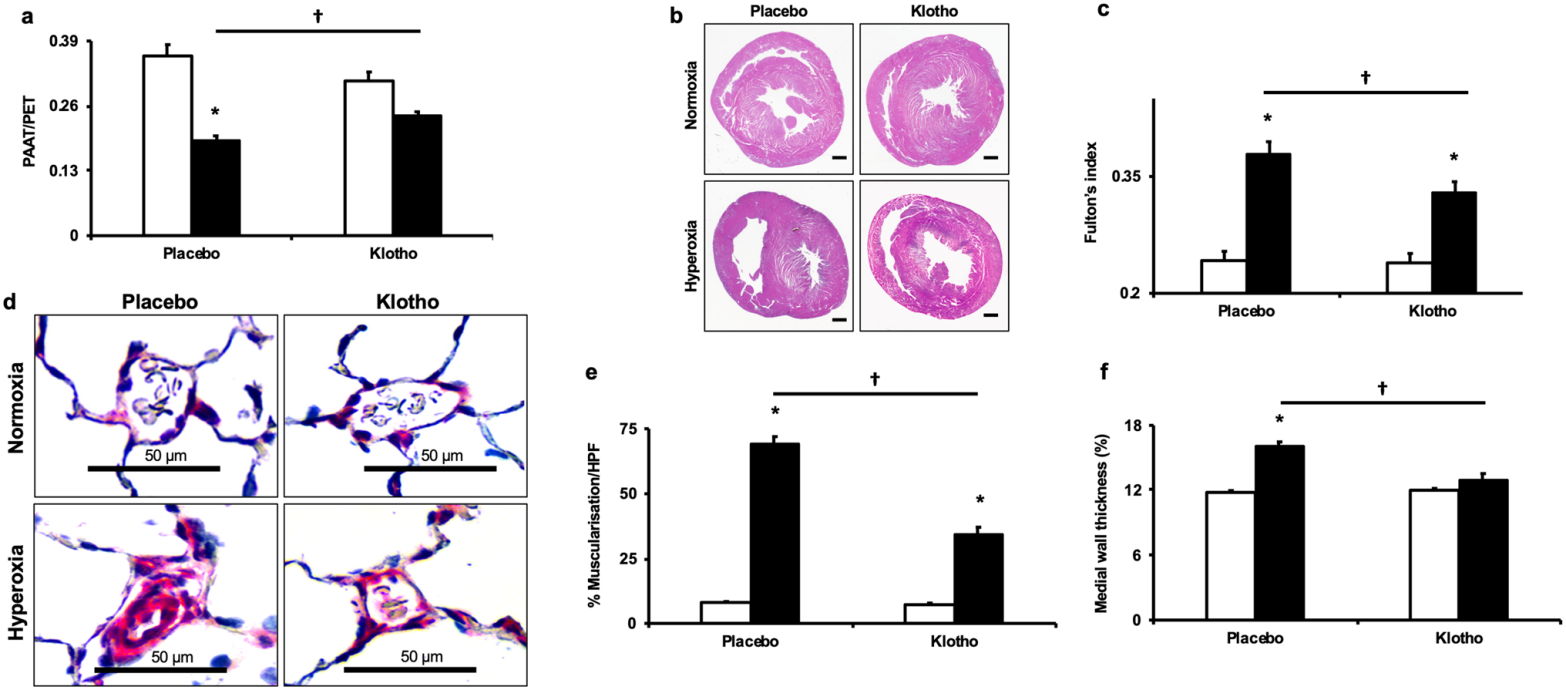
Klotho attenuates pulmonary hypertension and vascular remodeling. (**a**) Klotho treated hyperoxia-exposed rats had increased PAAT/PET ratio and hematoxylin and eosin staining of heart sections revealed (**b**) decreased RV and LV hypertrophy in treated rats. Original magnification × 10, Scale bars are 1 mm. Klotho supplementation also reduced (**c**) the weight ratio of the RV to LV + septum (Fulton’s Index) in hyperoxia exposed rats. (**d**) Lung sections stained with α-smooth muscle actin (red) demonstrating decreased vascular remodeling in hyperoxia-exposed rats treated with Klotho. Magnification × 40. Scale bars are 50 µm. Klotho supplementation significantly decreased (**e**) percent muscularization and (**f**) medial wall thickness of small pulmonary vessels (< 50 µm) in the hyperoxia-exposed animals (data are mean ± SEM; *P* < 0.05; * normoxia vs hyperoxia; † hyperoxia-PL vs hyperoxia-Klotho; N = 5–7/group). White bars indicate normoxia and black bars indicate hyperoxia.

In order to assess whether the improvement in angiogenesis following Klotho supplementation would be associated with reduced PH, the echocardiographic-derived PAAT/PET ratio, a surrogate measure of PH, was evaluated in the different animal groups. PL-treated hyperoxia exposed rats had decreased PAAT/PET (0.36 ± 0.02 vs 0.19 ± 0.01; normoxia-PL vs hyperoxia-PL; *P* < 0.05; N = 5/group) and this was significantly improved following early Klotho administration (0.19 ± 0.01 vs 0.24 ± 0.008; hyperoxia-PL vs hyperoxia-Klotho; *P* < 0.05; N = 5/group, Fig. 5a). Additionally, while PL-treated rats exposed to neonatal hyperoxia had increased RV/LV + S, the degree of right ventricular hypertrophy significantly improved following early administration of Klotho, (Fig. 5b,c).

Given that vascular remodeling is also a component of PH complicating BPD, we determined the effect of Klotho supplementation on pulmonary vascular remodeling. Lung sections were immunostained with vWF and smooth muscle actin antibodies. PL-treated hyperoxia exposed rats had increased vascular remodeling as evidenced by increased percentage of muscularized pulmonary vessels and greater medial wall thickness. These alterations were significantly improved following early administration of Klotho (Fig. 5d–f).

**Figure 6:**
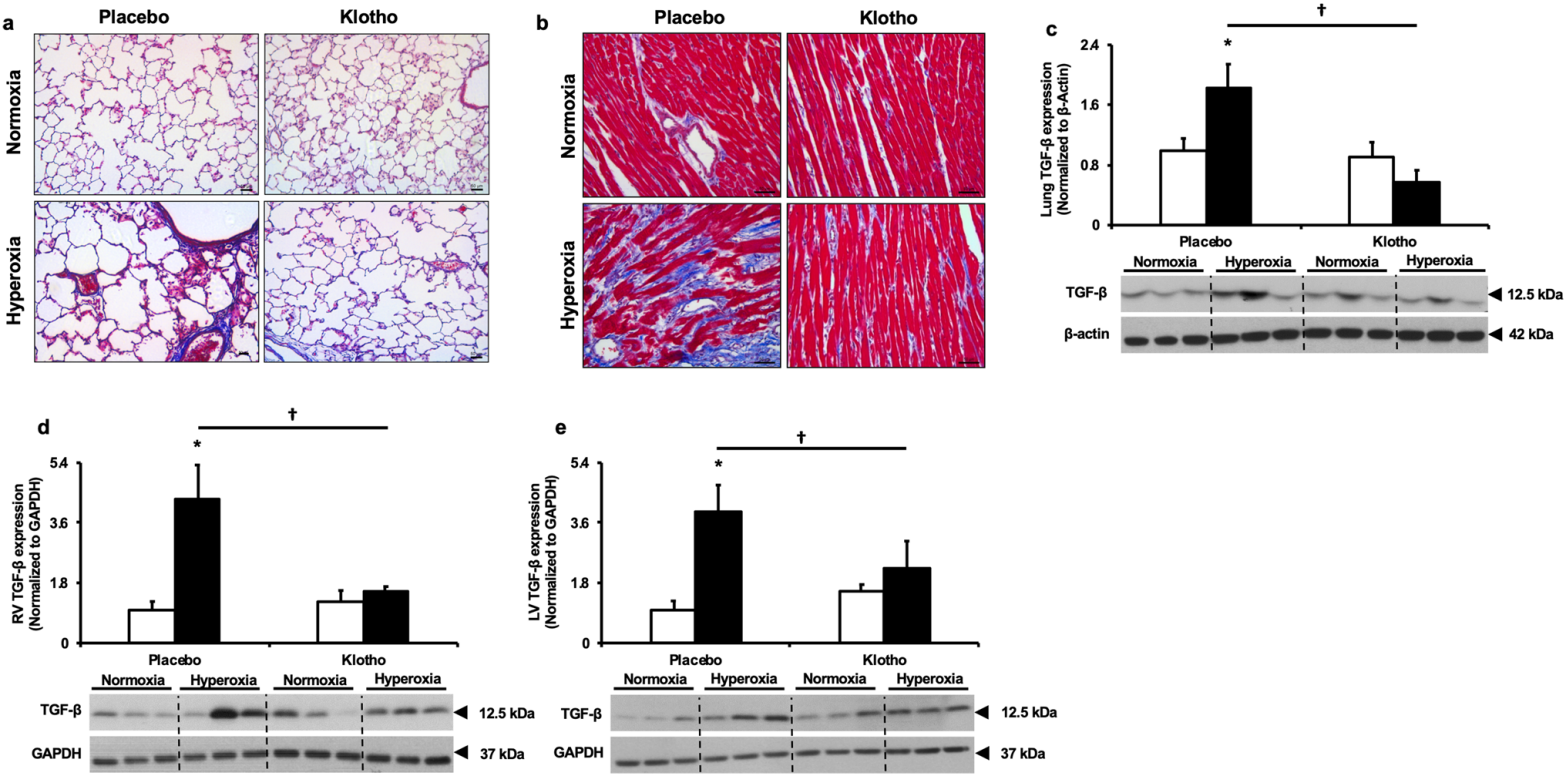
Klotho attenuates lung and heart fibrosis. Masson’s trichrome staining of (**a**) lung and (**b**) right ventricle sections demonstrating decreased fibrosis in hyperoxia-exposed Klotho treated rats. Original magnification × 20, Scale bars are 50 µm. Klotho supplementation in hyperoxia-exposed rats decreased (**c**) lung, (**d**) RV and (**e**) LV TGF-β expression. Representative Western blots are shown in the lower panels. Lung TGF-β expression is normalized to β-Actin. RV and LV TGF-β expression is normalized to GAPDH. White bars indicate normoxia, and black bars indicate hyperoxia (Data are mean ± SEM; *P* < 0.05; * normoxia-PL vs hyperoxia-PL; † hyperoxia-PL vs hyperoxia-Klotho; N = 5/group).

Predictably, hyperoxia-PL treated rats had increased lung and RV fibrosis (Fig. 6a,b) that were distinctly reduced in Klotho supplemented animals. Since Klotho deficient animals have increased TGF-β levels^33^ and TGF-β is known to play an important role in BPD–PH pathogenesis^34^, we evaluated the effect of Klotho on lung and RV TGF-β levels. Whereas hyperoxia-PL treated animals had increased lung and RV TGF-β expression, early Klotho supplementation in hyperoxia-exposed animals decreased lung, RV and LV TGF-β levels (Fig. 6c–e).

#### Early Klotho supplementation and cardiac function

Since cardiac dysfunction is a significant cause of mortality in preterm infants with BPD–PH, we evaluated the potential cardioprotective effects of Klotho. Tricuspid annular plane systolic excursion (TAPSE) is an echocardiographic measure of RV function. PL-treated hyperoxia exposed rats had evidence of RV dysfunction as demonstrated by shortening of TAPSE but early administration of Klotho did not significantly alter TAPSE in hyperoxia-exposed animals^35^. In contrast, whereas PL-treated neonatal hyperoxia-exposed rats had decreased LV ejection fraction (70 ± 1.5 vs 35 ± 2.6%; normoxia-PL vs hyperoxia-PL; *P* < 0.05; N = 3–5/group), and increased Tei index (0.6 ± 0.04 vs 1 ± 0.13; normoxia-PL vs hyperoxia-PL; *P* < 0.05; N = 3–5/group), Klotho administration significantly improved these measurements of left ventricular function (Table 2).

**Table 2:** Klotho and cardiac function.

	RA-PL	RA-Klotho	Hyperoxia-PL	Hyperoxia-Klotho
Heart rate (BPM)	407 ± 42	371 ± 6	391 ± 29	403 ± 19
TAPSE (mm)	2.3 ± 0.2	2.35 ± 0.2	1.87 ± 0.3	1.85 ± 0.3
RV anterior wall thickness (mm)	0.64 ± 0.3	0.64 ± 0.2	1.73 ± 0.3*	1.14 ± 0.2^†^
LV myocardial Performance index (Tei)	0.6 ± 0.1	0.6 ± 0.1	1.05 ± 0.2*	0.6 ± 0.05^†^
LV indexed mass	2.28 ± 0.5	2.54 ± 0.3	4.24 ± 1.2	2.76 ± 0.8
LV anterior wall thickness (mm)	1.4 ± 0.05	1.3 ± 0.01	1.8 ± 0.3*	1.35 ± 0.1^†^
LV ejection fraction (%)	70 ± 2	73 ± 2	35 ± 4*	51 ± 16^†^

*P* value < 0.05 *RA-PL vs Hyperoxia-PL, †Hyperoxia-PL vs Hyperoxia-Klotho.

#### Early Klotho supplementation reduces alveolar simplification

**Figure 7:**
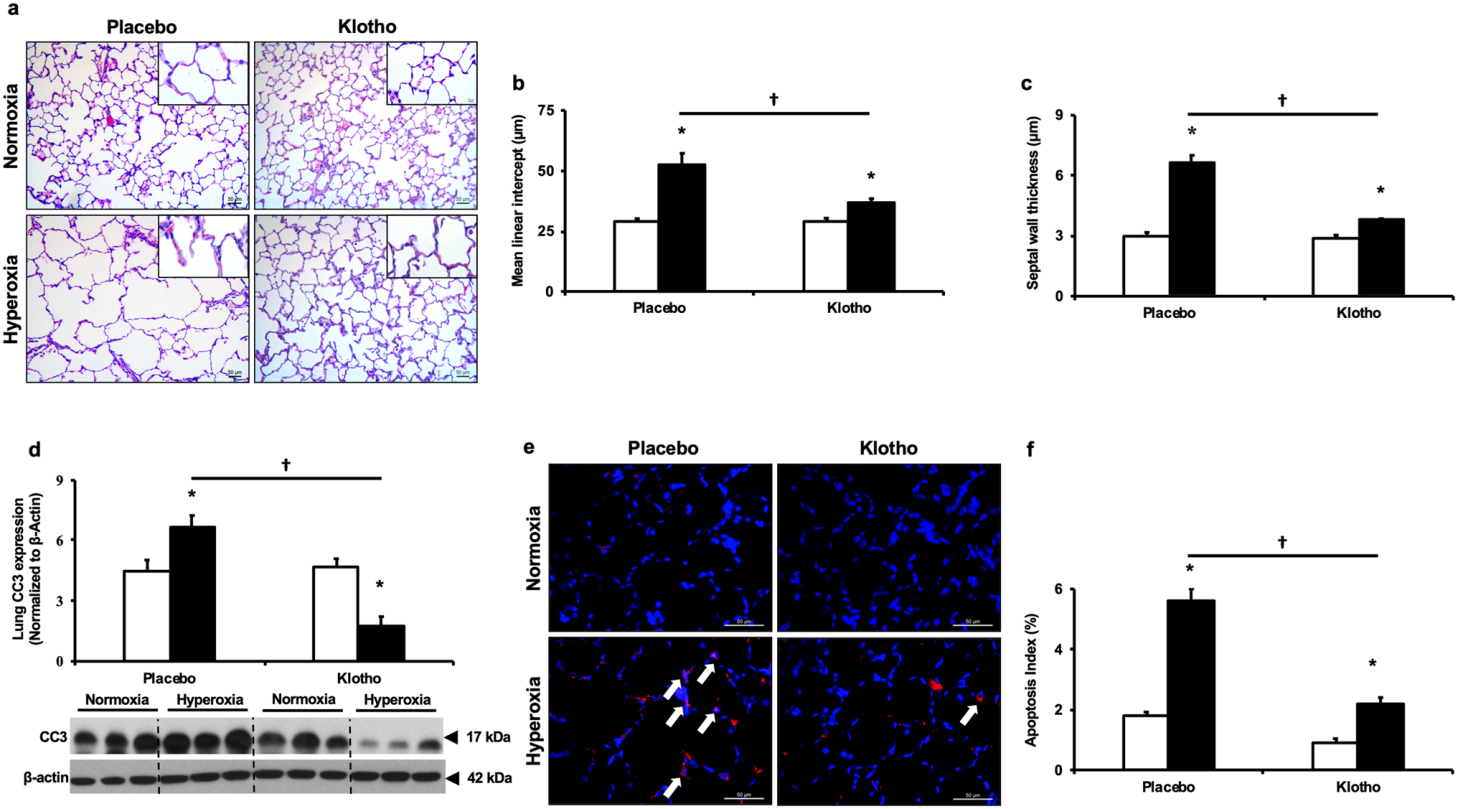
Klotho improves lung alveolarization. (**a**) Haematoxylin and eosin stained lung sections demonstrating improved alveolar structure in hyperoxia-exposed rats treated with Klotho. Original magnification × 20. Scale bars are 50 µm. Boxed panel is × 40. Histogram showing decreased (**b**) mean linear intercept (**c**) septal wall thickness and (**d**) cleaved caspase-3 protein expression (CC3) in Klotho treated hyperoxia-exposed rats. A representative Western blot is shown in the lower panel. CC3 is normalized to β-actin. (**e**) Lung sections stained with CC3 antibody (red) revealed decreased CC3^pos^ lung cells and reduced (**f**) lung apoptotic index in hyperoxia-exposed rats treated with recombinant Klotho (Data are mean ± SEM; *P* < 0.05; * normoxia vs hyperoxia; † hyperoxia-PL vs hyperoxia-Klotho; N = 5–7/group). White bars indicate normoxia, and black bars indicate hyperoxia.

Given that patients with BPD and PH exhibit arrest of alveolar growth, and that angiogenesis is closely intertwined with alveolar development, we assessed whether Klotho supplementation could also preserve alveolar structures. Whereas PL-treated neonatal hyperoxia exposed animals displayed impaired alveolar development as evidenced by large alveoli and decreased septation, early administration of Klotho improved alveolar structure, (Fig. 7a). Morphometric analysis revealed increased MLI (29 ± 1.4 vs 52 ± 5 µm; normoxia-PL vs hyperoxia-PL; *P* < 0.05; N = 6–10/group) and septal thickness in the hyperoxia PL-treated animals. Early Klotho supplementation significantly improved these measures of alveolarization (Fig. 7b,c).

In order to investigate alternative mechanisms by which Klotho improved lung structure, we also evaluated the effect of Klotho on lung apoptosis. Paraffin embedded lung sections were immunostained with polyclonal CC3 antibody and the number of CC3 positive cells in ten randomly selected fields was quantified. The apoptotic index was obtained by the formula: number of CC3 positive cells per field /total number of cells per field. PL-treated hyperoxia-exposed animals had increased apoptosis as evidenced by more CC3 positive cells/total number of nucleated lung cells and increased CC3 protein expression in lung homogenates. These abnormalities were significantly decreased in hyperoxia-exposed animals treated with Klotho (Fig. 7d–f).

#### Early Klotho supplementation reduces lung and myocardial oxidative stress

**Figure 8:**
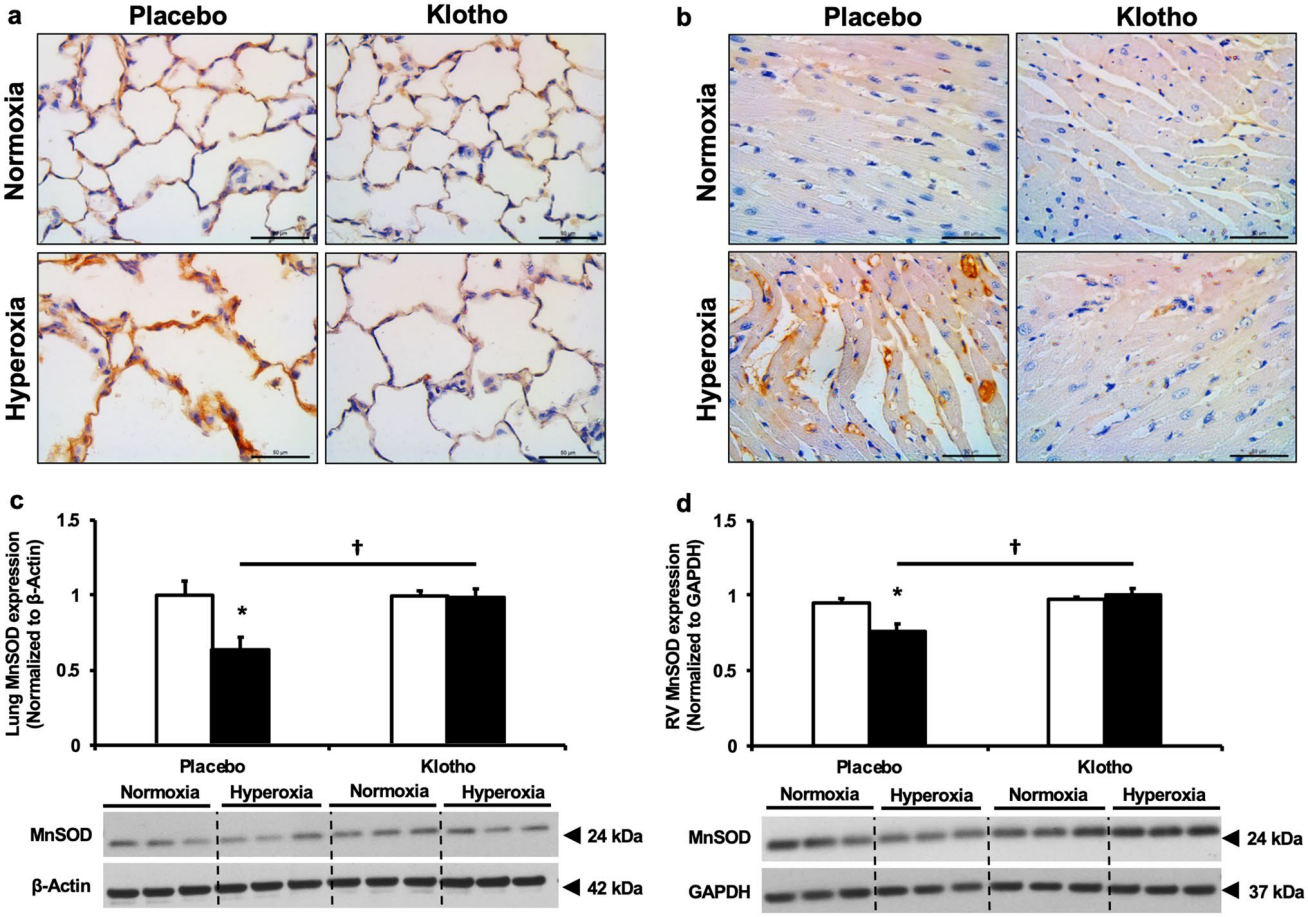
Early supplemental Klotho reduces lung and right ventricle (RV) oxidative stress. Decreased (**a**) lung and (**b**) RV 3-Nitrotyrosine (brown) immunostaining in hyperoxic rats who received supplemental Klotho. Original magnification × 40. Scale bars are 50 µm. Klotho supplementation increased (**c**) lung and (**d**) RV MnSOD expression. Representative western blots are shown in the lower panel. Lung MnSOD expression is normalized to β-Actin and RV MnSOD expression is normalized to GAPDH. White bars indicate normoxia, and black bars indicate hyperoxia (Data are mean ± SEM; *P* < 0.05; * normoxia-PL vs hyperoxia-PL; † hyperoxia-PL vs hyperoxia-Klotho; N = 3–5/group).

Since preterm infants have limited antioxidant capabilities, we evaluated the effect of supplemental Klotho on lung and RV oxidative stress in rats exposed to neonatal hyperoxia. Nitrotyrosine is marker of oxidative stress generated by chemical oxidation of tyrosine by peroxynitrite. This is known to be increased in plasma of preterm infants who develop BPD^36^. Whereas PL-treated hyperoxia exposed rats had greater lung and RV oxidative stress as evidenced by increased nitrotyrosine expression, this oxidative stress marker was reduced in Klotho supplemented rats, (Fig. 8a,b). Moreover, this improvement in oxidative stress was associated with a significant increase in MnSOD protein expression in lung and RV homogenates of hyperoxia-exposed Klotho supplemented animals (Fig. 8c,d). LV MnSOD expression was not significantly altered by hyperoxia or Klotho treatment.

#### Effect of early Klotho supplementation on growth and survival

Since infants with BPD complicated by PH grow poorly^2,37^, and Klotho deficiency is associated with poor growth, we evaluated the effect of early Klotho supplementation on growth of rats exposed to neonatal hyperoxia. Interestingly, neonatal hyperoxia exposure did not significantly alter body weight of 6 week old rats, (179 ± 6 vs 176 ± 8 g; normoxia-PL vs hyperoxia-PL; N = 7–15 /group) and there was no significant difference with Klotho treatment, (176 ± 8 vs 185 ± 9 g; hyperoxia-PL vs hyperoxia-Klotho; N = 7–11 group). In contrast, whereas the survival of normoxia treated rats was similar in both groups, (94% vs 94%, normoxia-PL vs normoxia-Klotho, N = 16/group), there was a trend for improved survival in the Klotho supplemented hyperoxia-exposed rats, which did not reach statistical significance (44% vs 69%; hyperoxia-PL vs hyperoxia-Klotho, N = 7–11/group).

## Discussion

Emerging evidence suggest that perturbation of key molecular aging pathways play a key role in BPD–PH pathogenesis^1^. Here, we provide the first published evidence demonstrating that soluble Klotho, an anti-aging protein with potent antioxidant, pro-angiogenic and anti-fibrotic properties, is reduced in the cord blood of preterm infants who subsequently develop BPD–PH. We also show, in a rodent model of severe BPD complicated by PH, that early Klotho supplementation improves lung vascular development, reduces PH, attenuates vascular remodeling and prevents long-term cardiac dysfunction, through antioxidant, pro-angiogenic and anti-fibrotic effects. Together, our present findings provide novel insight into the potential contribution of Klotho deficiency in BPD–PH pathogenesis and suggest a new target to improve long-term cardiopulmonary outcomes in preterm infants. This is particularly important, as there are currently few effective strategies to treat BPD and PH. Indeed, strategies targeting inflammation^38,39^, oxidative stress^40^ and angiogenesis^41^ have shown promise in preclinical models, but owing the multifactorial etiology of BPD and PH, few have been used clinically.

In our current study, we first show that decreased cord Klotho levels are associated with an increased risk for severe BPD and PH. Klotho is known to be secreted by the placenta and whereas cord Klotho levels are typically higher than adult levels, plasma Klotho concentration is greater in full term than preterm infants at 14 and 28 days of life^42^. We speculate that variations in Klotho circulating in cord blood at birth are heavily influenced by placental function (or dysfunction). This has been described in our recent publication^27^. Specifically, maternal vascular malperfusion (MVM), which is a histologic marker of upstream placental dysfunction arising during early placentation, is associated with decreased cord blood Klotho levels. Placental MVM is also prominent in extremely preterm births in which BPD-associated PH developed ^43^.

Neonatal hyperoxia exposure is another key factor in BPD and PH pathogenesis. In our present study, employing an established experimental model of BPD and PH, we also demonstrate a marked reduction in lung Klotho expression and circulating Klotho levels following neonatal hyperoxia exposure. Our findings extend those of other investigators who demonstrate reduced Klotho expression in hyperoxia-exposed alveolar epithelial cells ^17,44^ and they also importantly suggest that not only does placental insults lead to Klotho deficiency, but postnatal factors further reduce lung and circulating Klotho levels.

In order to ascertain whether early Klotho supplementation could reduce BPD and PH, we utilized a neonatal hyperoxia exposure rodent model of severe BPD–PH. Infants with BPD and PH exhibit decreased density of peripheral pulmonary arteries^45^ and there is substantial evidence linking altered pro-angiogenic signaling pathways to BPD and PH pathogenesis^46^. In our study, we not only demonstrate that Klotho improves viability, proliferation and capillary tube formation of hyperoxia-exposed HPAECs but early supplementation of Klotho increased the number of peripheral pulmonary vessels in neonatal hyperoxia-exposed rodents. Our findings are consistent with other studies, which demonstrated improved tube formation, and survival of hydrogen peroxide injured Klotho-treated human umbilical venous cells^19^. Moreover, although the mechanisms by which Klotho regulates endothelial function and angiogenesis are still under investigation, emerging evidence suggest that the vasculoprotective effects of soluble Klotho may be secondary to its regulation of VEGFR2/transient receptor potential canonical-1 mediated Ca^2+^ influx^22^, modulation of endothelial nitric oxide production^47,48^ and its anti-oxidant properties. We speculate that Klotho’s direct pulmonary vascular protective properties in our study are secondary to its antioxidant effects and decreased TGF-β levels. This latter finding is supported by prior studies showing that TGF-β via its activation of the Smad 2/3 signaling pathway negatively regulates angiogenesis^49^.

Another important finding in our study is the significant reduction in vascular remodeling evidenced in neonatal hyperoxia-exposed rodents who received early Klotho supplementation. Other studies have demonstrated that Klotho-overexpressing mesenchymal stem cells reduce vascular remodeling in rodents with monocrotaline-induced PH by reducing inflammation, and increasing endothelial nitric oxide synthase levels^50^. Additionally, Klotho inhibits renal fibrosis by antagonizing Wnt/β-catenin signaling^51^. While these mechanisms may contribute to the effects of Klotho evidenced in our study, we postulate that our current findings are secondary to decreased TGF-β pro-fibrotic signaling.

Cardiac dysfunction is a significant cause of mortality in preterm patients with BPD and PH^52^. Along with RV dysfunction, infants also develop LV dysfunction often secondary to septal bowing, myocardial ischemia and toxic effects of hyperoxia on the developing myocardium. Surprisingly, in our current study, while early Klotho supplementation significantly improved LV function, there were no significant changes in TAPSE, a measure of RV function. We speculate that with improvement in PH, the ventricular septal position shifts, allowing for volumetric changes in the left ventricle and improvement in the ejection fraction. RV function also impacts LV function through ventricular interdependence such that changes in RV geometry/function may also impact LV function. Importantly, TAPSE is only one measure of RV function, representing the RV longitudinal function, and it does not account for other aspects of RV function including circumferential shortening. Echocardiographic measures of RV function have always been limited owing to the complex geometry of the right ventricle, thus it is possible that there may have been improvement in the RV function, which was not accounted for by TAPSE. Our current finding of improved LV function is nonetheless in keeping with other investigators who demonstrated improved cardiac function in mice with doxorubicin-induced cardiac injury following Klotho administration^53^.

In our present study, lungs from all experimental groups were also uniformly inflated in order to assess the effects of Klotho on lung alveolarization. Klotho supplemented hyperoxia-exposed rats had a significant improvement in alveolar structure. Lung vascular development is closely linked with alveolarization and impaired pulmonary function is seen in preterm survivors of BPD–PH even into adulthood^54,55^. While it is plausible that the improvement in alveolarization in treated animals may be related to increased lung angiogenesis, we speculate that our finding of decreased apoptosis in the treated animals may have contributed to this preservation of alveolar structure. This reduced apoptosis in the treated animals may be due to decreased oxidative stress or maybe related to decreased TGF-β related pro-apoptotic pathways.

The present study has several limitations. While cord blood Klotho was decreased with MVM in the current study (data not shown), the much smaller sample size included here did not allow us to comprehensively analyze the interactions between Klotho, MVM and BPD–PH outcomes collectively. The role of the placenta in mediating BPD–PH via Klotho cannot be determined by the clinical observations alone in this study, but combined with the animal findings presented here, these potential interactions are supported. Further studies incorporating larger infant cohorts and animal models of placental dysfunction are warranted. Also, while there are currently no clear genetic links to low Klotho levels in cord blood, investigation of genetic factors that influence Klotho is also warranted. The BPD–PH rodent model used was also severe and may not reflect the disease evidenced in many preterm survivors. In addition, while exposure to high oxygen levels is important in BPD–PH pathogenesis, other factors including intermittent hypoxia, pre-and postnatal exposure to infection, mechanical ventilation and poor nutrition, contribute to this disease. It is therefore possible that the additive effects of these insults on the preterm cardiopulmonary system will potentially alter the efficacy of Klotho. We also exposed the rat pups to a high oxygen concentration from birth to postnatal day 21, which corresponds to the saccular-alveolar stage of lung development. Most preterm infants at highest risk for BPD are born in the cannalicular and saccular stages of lung development, with repair and recovery occurring in the alveolar phase. Further dose–response studies will also need to be performed, as early Klotho supplementation did not return lung structure or cardiac function to those observed under normoxic conditions. It will also be crucial to perform studies evaluating the effect of later administration of Klotho in established neonatal PH models. Moreover, since Klotho affects calcium and phosphorus metabolism, it will be important to follow calcium and phosphorus levels longitudinally.

In conclusion, to the best of our knowledge, this is the first study which evaluates cord blood Klotho levels in preterm infants who develop BPD and PH. We show that Klotho deficiency predicts BPD and PH risk in preterm infants. Moreover, through anti-oxidant, anti-fibrotic, anti-apoptotic and pro-angiogenic mechanisms, early Klotho supplementation improves lung structure, attenuates vascular remodeling and reduces cardiac hypertrophy and dysfunction in rats with experimental severe BPD complicated by PH. Together, our findings suggest that Klotho deficiency is a potential mediator and biomarker for BPD and PH risk and early interventions, which augment Klotho levels, may be an effective strategy to improve cardiopulmonary outcomes in preterm infants. Further studies will be needed evaluating the effects of Klotho on other developing organ systems as this will be crucial to translate the findings of our studies to the bedside.

## Supplementary information

Supplementary information.

## Data Availability

The datasets generated during and/or analyzed during the current study are available from the corresponding author on reasonable request.
