# Supplementary material for: Soluble Klotho, a biomarker and therapeutic strategy to reduce bronchopulmonary dysplasia and pulmonary hypertension in preterm infants
Source: Sci Rep. 2020 Jul 23;10:12368. doi: 10.1038/s41598-020-69296-1 (PMC7378054; doi:10.1038/s41598-020-69296-1)

Soluble Klotho, a Biomarker and Therapeutic Strategy to Reduce Bronchopulmonary Dysplasia and Pulmonary Hypertension in Preterm Infants

Sunil Batlahally[1,2], Andrew Franklin[5], Andreas Damianos[1,2], Jian Huang[1,2], Pingping Chen[1,2], Mayank Sharma[1,2], Joanne Duara[1,2], Divya Keerthy[1,2], Ronald Zambrano[1,2], Lina Shehadeh[3], Eliana C. Martinez[3], Marissa J DeFreitas[1], Shathiyah Kulandavelu[1,3], Carolyn L Abitbol[1], Michael J Freundlich[1], Rosemeire M Kanashiro-Takeuchi[1,3], Augusto Schmidt[1,2], Merline Benny[1,2], Shu Wu[1,2], Karen K. Mestan[5*] and Karen C. Young[1,2,3*]

Running Title: Klotho, bronchopulmonary dysplasia and pulmonary hypertension

[1]Department of Pediatrics, University of Miami Miller School of Medicine, Miami, FL

[2]Batchelor Children's Research Institute, University of Miami Miller School of Medicine, Miami, FL

[3]The Interdisciplinary Stem Cell Institute, University of Miami Miller School of Medicine, Miami, FL

[4] Department of Molecular and Cellular Pharmacology, University of Miami Miller School of Medicine, Miami, FL

[5]Ann & Robert H. Lurie Children's Hospital of Chicago, Northwestern University Feinberg School of Medicine, Chicago, IL

*Karen K. Mestan and Karen C. Young are co-senior authors.

Figure 1a

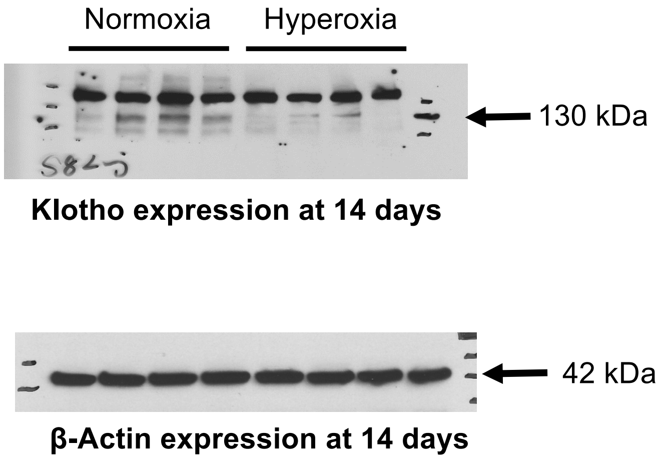

Figure 1b

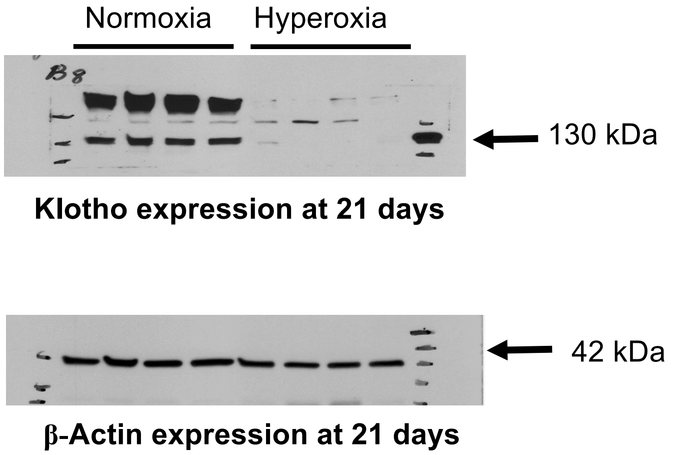

Figure 2a

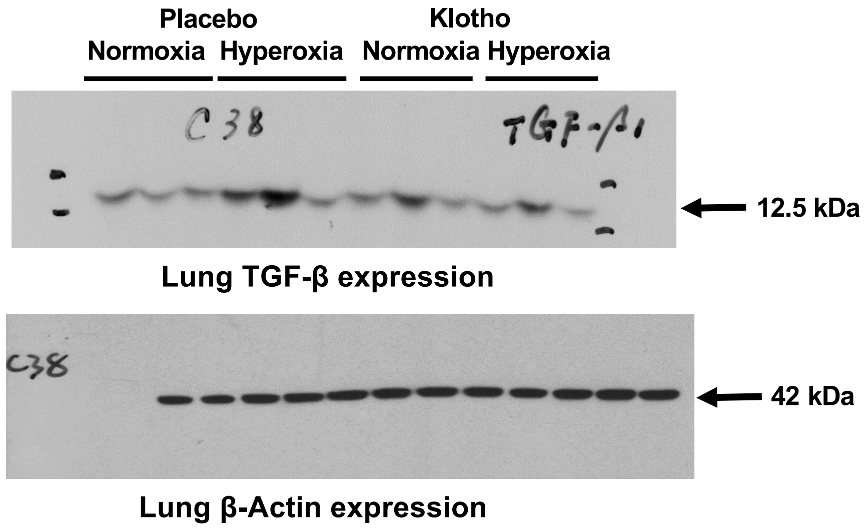

Figure 2b

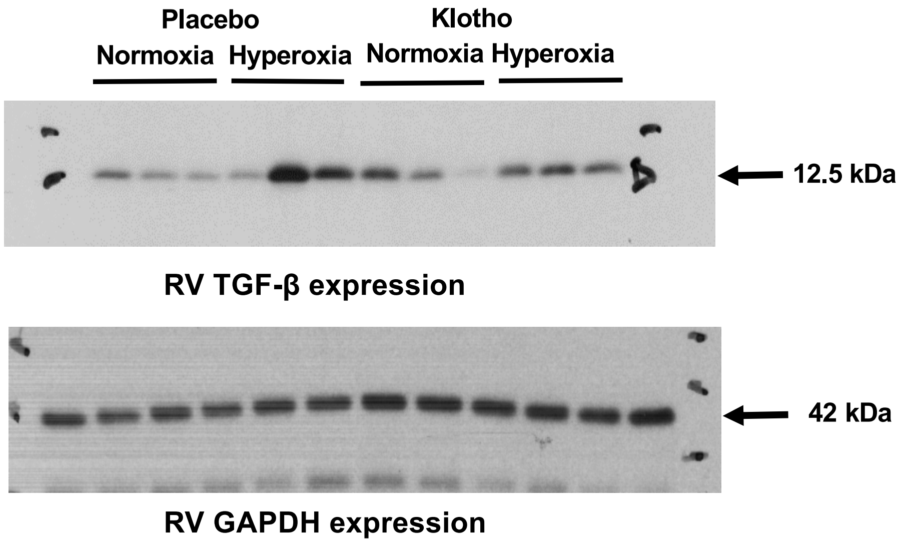

Figure 3a

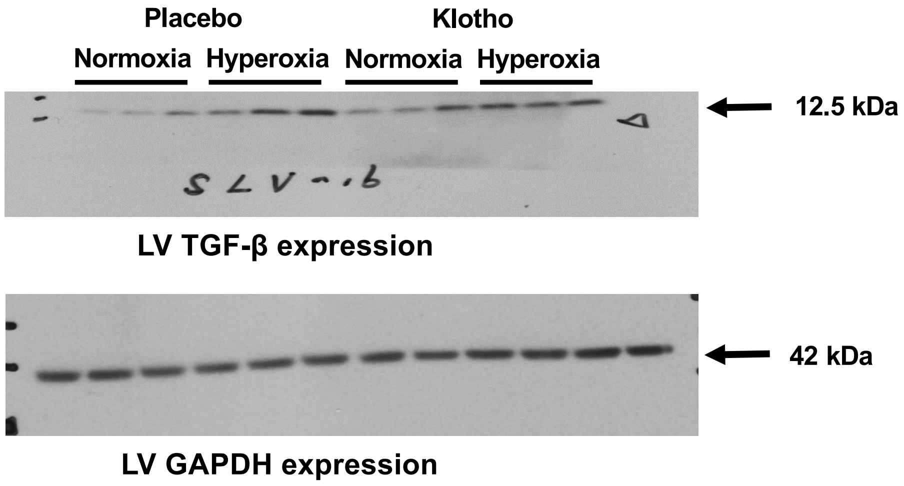

Figure 3b

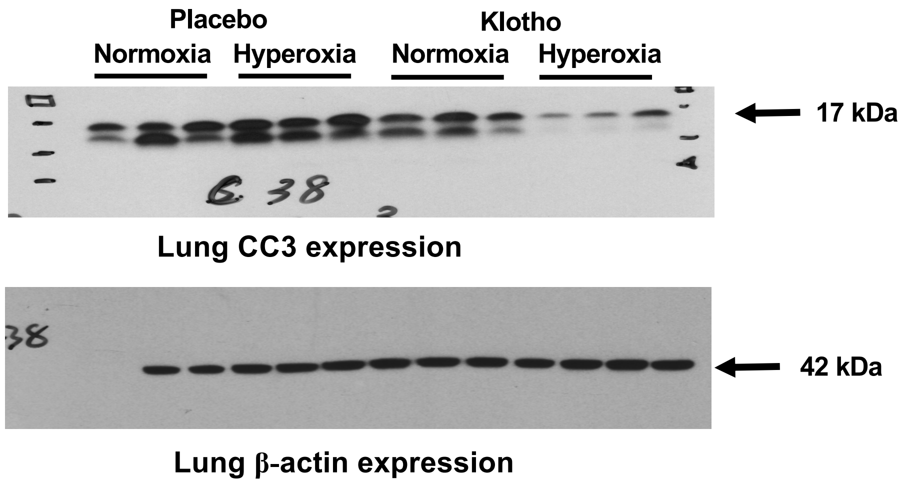

Figure 4a

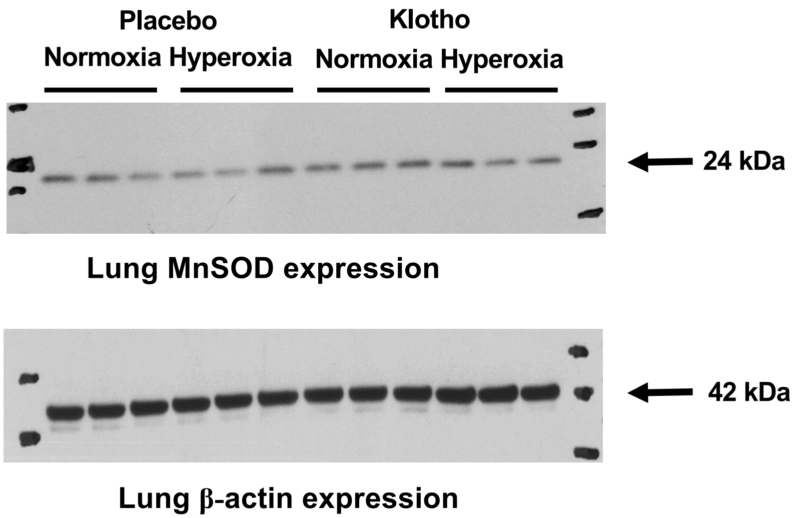

Figure 4b

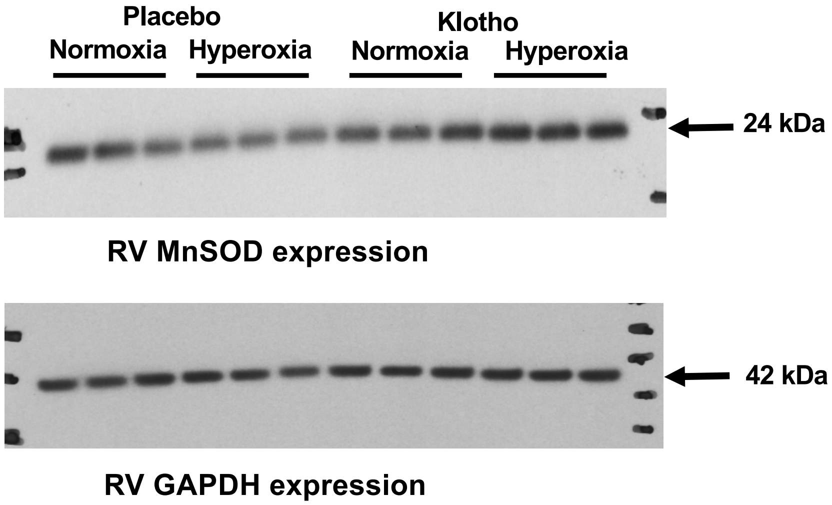

Supplement: Supplementary file 1 — Supplementary information. [file 41598_2020_69296_MOESM1_ESM.pdf]
